# Targeted Tuberculosis (TB) Vaccination Strategies in the United States: A Modeling Study

**DOI:** 10.64898/2026.05.11.26352914

**Published:** 2026-05-14

**Authors:** Jessica E. Rothman, Kenneth G. Castro, Benjamin A. Lopman, Neel R. Gandhi, Kristin N. Nelson

## Abstract

**Background:**

Tuberculosis (TB) incidence in the United States has remained elevated above pre-pandemic levels since 2021, with over 85% of cases resulting from reactivation of *Mycobacterium tuberculosis* ( *Mtb* ) infection. New vaccines that would prevent TB in adults are under development, but the potential health impact of a program prioritizing non-U.S.-born persons and persons with medical comorbidities, including persons living with HIV (PLWH), has not been evaluated.

**Methods:**

We developed a deterministic compartmental transmission model that simulates *Mtb* infection, transmission, and progression to TB in the U.S., both in the general population and in key high-risk groups. We calibrated the model to 2024 U.S. TB surveillance data and estimated annual cases prevented, percent reduction in annual TB cases, and number needed to vaccinate (NNV, a measure of vaccine program efficiency) at equilibrium conditions for targeted vaccination strategies under optimistic and plausible scenarios, varying assumptions of vaccine efficacy, duration of protection, and achieved vaccination coverage in high-risk groups.

**Findings:**

Under an optimistic scenario, vaccinating PLWH, non-U.S.-born persons, and persons with medical comorbidities (all high-risk groups) prevented 5,385 cases per year (51·8% reduction, NNV = 366). Under a more conservative plausible scenario, the same strategy prevented 1,348 cases per year (13·0% reduction, NNV = 510). The efficiency and impact of targeting strategies we considered were preserved across all sensitivity and uncertainty analyses.

**Interpretation:**

Targeted vaccination of persons with *Mtb* infection in population subgroups recognized to be at high-risk for TB can reduce incidence substantially. Strategies that include non- U.S.-born persons and PLWH are most efficient and impactful.

**Funding:**

American Lung Association, U.S. National Institutes of Health, and the Ferguson Fellowship.

## Full Text Availability

The license terms selected by the author(s) for this preprint version do not permit archiving in PMC. The full text is available from the preprint server.

